# Supplementary material for: Tracking B Cell Memory to SARS-CoV-2 Using Rare Cell Analysis System
Source: Vaccines (Basel). 2023 Mar 26;11(4):735. doi: 10.3390/vaccines11040735 (PMC10145117; doi:10.3390/vaccines11040735)
Supplement: Supplementary file 1 [file vaccines-11-00735-s001.zip › vaccines-2249851-supplementary.pdf]

**Table S1. Anti-spike-RBD binding antibody validation data by ELISA**

| Specimen Basic Information | | Raw Data | | | | Results | |
|---|---|---|---|---|---|---|---|
| Specimen | Concentration | Dilution | 450 nm | 620 nm | 450-620 nm | Diluted BAU/mL | BAU/mL |
| Negative control | - | - | 0.000 | 0.000 | 0.000 | - | - |
| WHO IS 20/136 -01 | 80 BAU/mL | - | 1.300 | 0.000 | 1.300 | - | - |
| WHO IS 20/136 -02 | 40 BAU/mL | - | 1.284 | 0.000 | 1.284 | - | - |
| WHO IS 20/136 -03 | 20 BAU/mL | - | 1.208 | 0.001 | 1.207 | - | - |
| WHO IS 20/136 -04 | 10 BAU/mL | - | 1.050 | 0.001 | 1.049 | - | - |
| WHO IS 20/136 -05 | 5 BAU/mL | - | 0.981 | 0.000 | 0.981 | - | - |
| WHO IS 20/136 -06 | 2.5 BAU/mL | - | 0.907 | 0.001 | 0.906 | - | - |
| WHO IS 20/136 -07 | 1.25 BAU/mL | - | 0.669 | 0.000 | 0.669 | - | - |
| WHO IS 20/136 -08 | 0.625 BAU/mL | - | 0.389 | 0.001 | 0.388 | - | - |
| WHO IS 20/150 | High | 1:400 | 0.818 | 0.000 | 0.818 | 2.077 | 830.8 |
| WHO IS 20/148 | Mid | 1:100 | 0.905 | 0.000 | 0.905 | 2.407 | 240.7 |
| WHO IS 20/142 | Negative control | 1:400 | 0.047 | 0.000 | 0.047 | <0.625 | - |
| WHO IS 20/136 -09 | 2.5 BAU/mL | - | 1.141 | 0.002 | 1.139 | - | - |
| WHO IS 20/136 -10 | 1.25 BAU/mL | - | 0.670 | 0.002 | 0.668 | - | - |
| WHO IS 20/136 -11 | 0.625 BAU/mL | - | 0.398 | 0.000 | 0.398 | - | - |
| 20/150 | High | 1:400 | 1.150 | 0.002 | 1.148 | 2.504 | 1001.47 |
| 20/148 | Mid | 1:100 | 0.987 | 0.001 | 0.986 | 2.093 | 209.28 |
| 20/142 | Negtive control | 1:400 | 0.023 | 0.000 | 0.023 | <0.625 | - |
| Negative control | - | - | 0.002 | 0.000 | 0.002 | - | - |
| WHO IS 20/136 -12 | 2.5 BAU/mL | - | 0.968 | -0.007 | 0.975 | - | - |
| WHO IS 20/136 -13 | 1.25 BAU/mL | - | 0.663 | -0.007 | 0.67 | - | - |
| WHO IS 20/136 -14 | 0.625 BAU/mL | - | 0.375 | -0.006 | 0.381 | - | - |
| 20/150 | High | 1:400 | 0.862 | 0.002 | 0.861 | 2.063 | 825.07 |
| 20/148 | Mid | 1:100 | 0.817 | 0.000 | 0.817 | 1.92 | 191.97 |
| 20/142 | Negtive control | 1:400 | 0.109 | -0.002 | 0.111 | <0.625 | - |

**Table S2. Anti-spike-RBD neutralizing antibody validation data by ELISA**

| Specimen Basic Information | | Raw Data | | | | Results | |
|---|---|---|---|---|---|---|---|
| Specimen | Concentration | Dilution | 450 nm | 620 nm | 450-620 nm | Diluted IU/mL | IU/mL |
| Negative control | - | - | 1.594 | 0.000 | 1.594 | - | - |
| WHO IS 20/136 -01 | 80 IU/mL | - | 0.029 | 0.000 | 0.029 | - | - |
| WHO IS 20/136 -02 | 40 IU/mL | - | 0.566 | 0.000 | 0.566 | - | - |
| WHO IS 20/136 -03 | 20 IU/mL | - | 0.873 | 0.000 | 0.873 | - | - |
| WHO IS 20/136 -04 | 10 IU/mL | - | 1.045 | 0.000 | 1.045 | - | - |
| WHO IS 20/136 -05 | 5 IU/mL | - | 1.278 | 0.000 | 1.278 | - | - |
| WHO IS 20/136 -06 | 2.5 IU/mL | - | 1.365 | 0.000 | 1.365 | - | - |
| WHO IS 20/136 -07 | 1.25 IU/mL | - | 1.541 | 0.000 | 1.541 | - | - |
| WHO IS 20/136 -08 | 0.625 IU/mL | - | 1.599 | 0.001 | 1.598 | - | - |
| WHO IS 20/150 | High | 1:100 | 0.982 | 0.000 | 0.982 | 13.64 | 1364 |
| WHO IS 20/148 | Mid | 1:20 | 0.923 | 0.000 | 0.923 | 17.21 | 344.2 |
| WHO IS 20/142 | Negative control | 1:100 | 1.561 | 0.000 | 1.561 | <10 | - |
| Negative control | - | - | 1.59 | -0.01 | 1.60 | - | - |
| WHO IS 20/136 -09 | 40 IU/mL | - | 0.28 | -0.01 | 0.29 | - | - |
| WHO IS 20/136 -10 | 20 IU/mL | - | 0.81 | -0.01 | 0.82 | - | - |
| WHO IS 20/136 -11 | 10 IU/mL | - | 1.08 | -0.01 | 1.09 | - | - |
| 20/150 | High | 1:100 | 1.04 | -0.01 | 1.05 | 12.012 | 1201.2 |
| 20/148 | Mid | 1:10 | 0.74 | -0.01 | 0.75 | 23.206 | 232.057 |
| 20/148 | Mid | 1:15 | 1.02 | -0.01 | 1.03 | 12.538 | 188.063 |
| 20/142 | Negtive control | 1:100 | 1.69 | -0.01 | 1.70 | <10 | - |
| Negative control | - | - | 1.84 | 0.00 | 1.84 | - | - |
| WHO IS 20/136 -12 | 40 IU/mL | - | 0.54 | 0.00 | 0.54 | - | - |
| WHO IS 20/136 -13 | 20 IU/mL | - | 1.12 | 0.00 | 1.12 | - | - |
| WHO IS 20/136 -14 | 10 IU/mL | - | 1.48 | 0.00 | 1.48 | - | - |
| 20/150 | High | 1:100 | 1.29 | 0.00 | 1.29 | 15.458 | 1545.81 |
| 20/148 | Mid | 1:10 | 1.15 | 0.00 | 1.15 | 20.039 | 200.387 |
| 20/148 | Mid | 1:15 | 1.38 | 0.00 | 1.38 | 12.435 | 186.532 |
| 20/142 | Negtive control | 1:100 | 1.72 | 0.00 | 1.72 | <10 | - |
| Negative control | - | - | 2.28 | 0.01 | 2.27 | - | - |
| WHO IS 20/136 -15 | 40 IU/mL | - | 0.83 | 0.04 | 0.79 | - | - |
| WHO IS 20/136 -16 | 20 IU/mL | - | 1.51 | 0.00 | 1.51 | - | - |
| WHO IS 20/136 -17 | 10 IU/mL | - | 2.00 | 0.01 | 1.99 | - | - |
| 20/150 | High | 1:100 | 1.62 | 0.01 | 1.61 | 18.787 | 1878.68 |
| 20/148 | Mid | 1:10 | 1.22 | 0.01 | 1.21 | 28.82 | 288.201 |
| 20/148 | Mid | 1:15 | 1.62 | 0.00 | 1.62 | 18.682 | 280.228 |
| 20/142 | Negtive control | 1:100 | 2.00 | 0.00 | 2.00 | <10 | - |

**Table S3. Demographic Information for vaccinated SARS-CoV-2 naïve subjects**

Number of individuals, n (%)

| | | | | |
|---|---|---|---|---|
| Prime dose | AZD1222 | BNT162b2 | mRNA-1273 | AZD1222 |
| Second dose | AZD1222 | BNT162b2 | mRNA-1273 | mRNA-1273 |
| Total number | n= 14 | n= 10 | n= 8 | n= 3 |
| Male | 9 (64.3%) | 6 (60.0%) | 6 (75.0%) | 3 (100.0%) |
| Female | 5 (35.7%) | 4 (40.0%) | 2 (25.0%) | 0 (0%) |

**Table S4. Reagents used for ELISA and spike-RBD specific memory B cells detection**

| Reagent | Vendor | Identifier | Concentiation / dilution |
|---|---|---|---|
| **Part I: Neutralizing antibody ELISA assay** | | | |
| ELISA Buffer kit | TONBO biosciences | 41-9245-KIT | |
| Recombinant SARS-CoV-2 Spike RBD | R&D Systems | 10500-CV-100 | 100 ng |
| ACE2 (Human) Recombinant Protein | Abnova | P6639 | 50 ng |
| LYNX Rapid HRP antibody conjugation kit | BIO-RAD | LNK006P | 40 μg |
| WHO International Standard for anti-SARS-CoV-2 immunoglobulin | NIBSC | 20/136 | 1000 IU/mL |
| WHO International Reference Panel for anti-SARS-CoV-2 immunoglo | NIBSC | 20/268 | |
| **Part II: Binding antibody ELISA assay** | | | |
| ELISA Buffer kit | TONBO biosciences | 41-9245-KIT | |
| Recombinant SARS-CoV-2 Spike RBD | R&D Systems | 10500-CV-100 | 100 ng |
| HRP Donkey anti-human IgG | BioLegend | 410902 | 1:20000 |
| Anti-SARS-CoV-2 Spike Glycoprotein S1 antibody CR3022 | abcam | ab273073 | 10 μg/mL, 1:10 |
| WHO International Standard for anti-SARS-CoV-2 immunoglobulin | NIBSC | 20/136 | 1000 BAU/mL |
| WHO International Reference Panel for anti-SARS-CoV-2 immunoglo | NIBSC | 20/268 | |
| **Part III: Spike-RBD specific cell dectecting** | | | |
| Recombinant SARS-CoV-2 Spike RBD | R&D Systems | 10500-CV-100 | 0.5 μg |
| Recombinant SARS-CoV-2 B.1.1.529 Spike RBD | R&D Systems | 11056-CV-100 | 0.5 μg |
| DAPI | | | 1:50 |
| PerCP anti-human CD19 | BioLegend | 392510 | 1:30 |
| FITC anti-human CD27 | BioLegend | 356404 | 1:30 |
| Rabbit Anti-Human IgG | Jackson ImmunoResearch | 309-001-008 | 1:100 |
| Qdot800 anti-Rabbit IgG | Invitrogen | Q-11471MP | 1:100 |
| APC anti-human CD3 | BioLegend | 317318 | 1:50 |
| DyLight 550 Conjugation Kit | abcam | AB201800-3X10UG | |

**Figure S1**

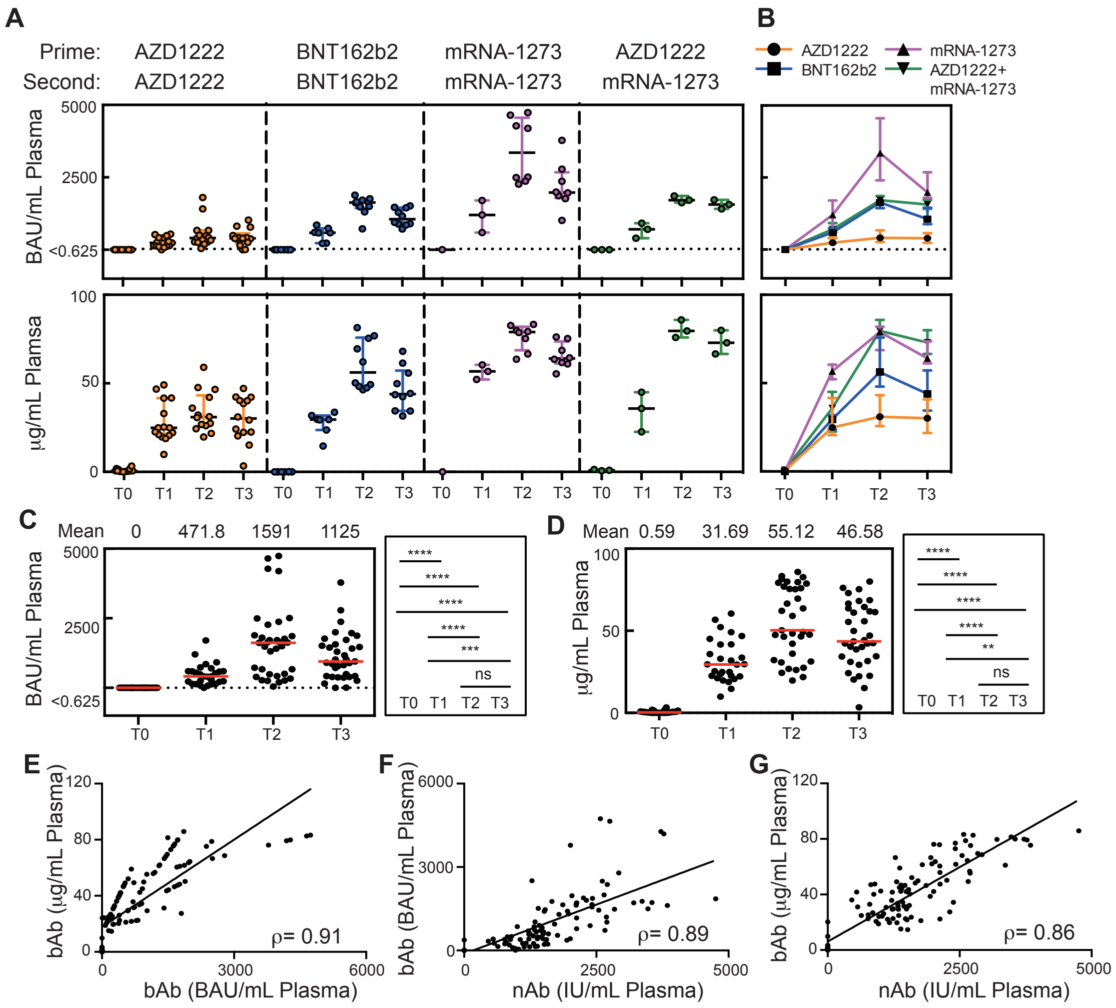
A
Prime:
AZD1222
BNT162b2
mRNA-1273
AZD1222
Second:
AZD1222
BNT162b2
mRNA-1273
mRNA-1273
BAU/mL Plasma
5000
2500
<0.625
μg/mL Plamsa
100
50
0
T0 T1 T2 T3 T0 T1 T2 T3 T0 T1 T2 T3 T0 T1 T2 T3
B
AZD1222
BNT162b2
mRNA-1273
AZD1222+ mRNA-1273
T0 T1 T2 T3
C
Mean 0 471.8 1591 1125
BAU/mL Plasma
5000
2500
<0.625
T0 T1 T2 T3
****
****
****
****
***
ns
T0 T1 T2 T3
D
Mean 0.59 31.69 55.12 46.58
μg/mL Plasma
100
50
0
T0 T1 T2 T3
****
****
****
****
**
ns
T0 T1 T2 T3
E
bAb (μg/mL Plasma)
120
80
40
0
ρ= 0.91
0 3000 6000
bAb (BAU/mL Plasma)
F
bAb (BAU/mL Plasma)
6000
3000
0
ρ= 0.89
0 2500 5000
nAb (IU/mL Plasma)
G
bAb (μg/mL Plasma)
120
80
40
0
ρ= 0.86
0 2500 5000
nAb (IU/mL Plasma)

**Figure S2**

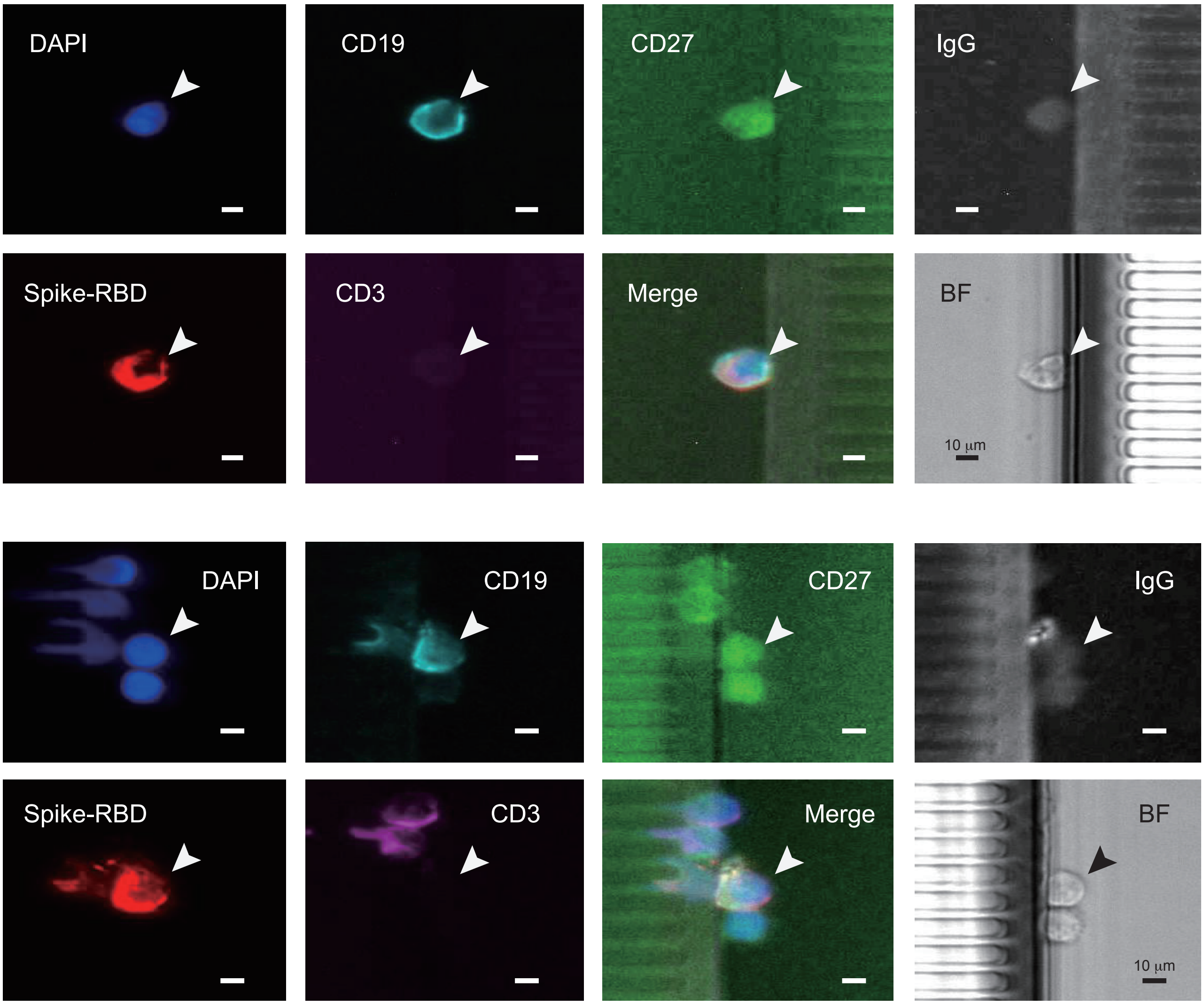
DAPI
CD19
CD27
IgG
Spike-RBD
CD3
Merge
BF
10 μm
DAPI
CD19
CD27
IgG
Spike-RBD
CD3
Merge
BF
10 μm

**Figure S3**

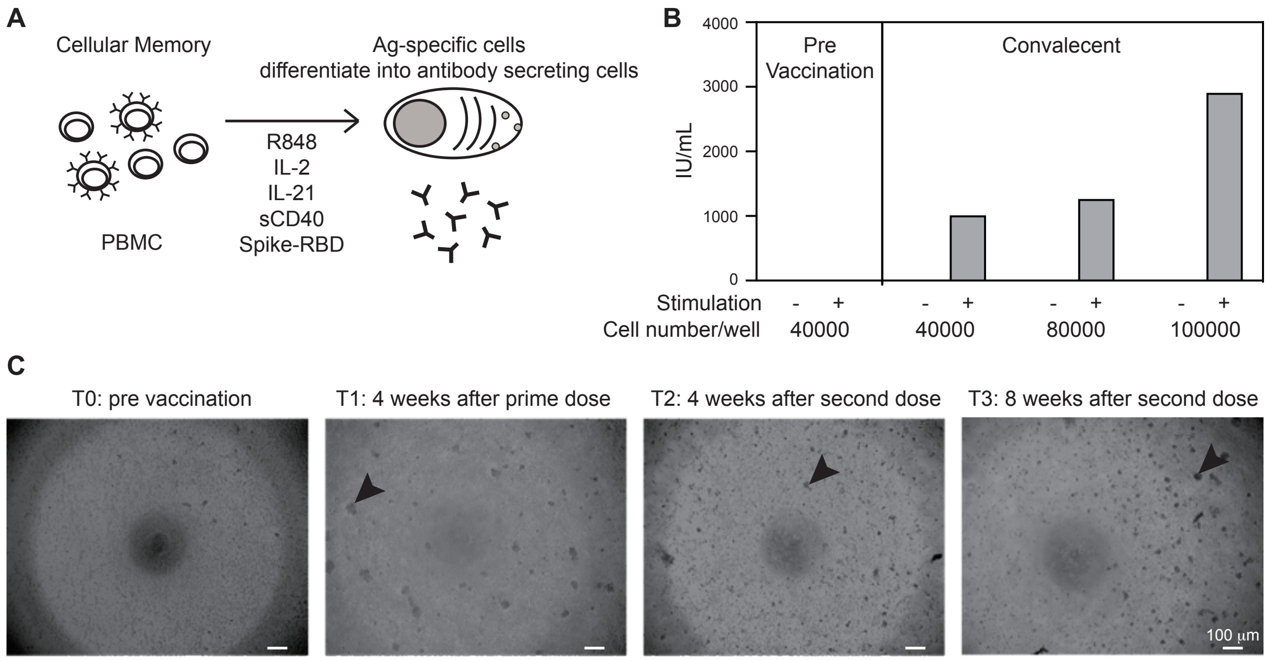
A
Cellular Memory
Ag-specific cells
differentiate into antibody secreting cells
R848
IL-2
IL-21
sCD40
Spike-RBD
PBMC
B
4000
3000
2000
1000
0
IU/mL
Pre
Vaccination
Convalecent
Stimulation - + - + - + - +
Cell number/well 40000 40000 80000 100000
C
T0: pre vaccination
T1: 4 weeks after prime dose
T2: 4 weeks after second dose
T3: 8 weeks after second dose
100 μm

**Figure S4**

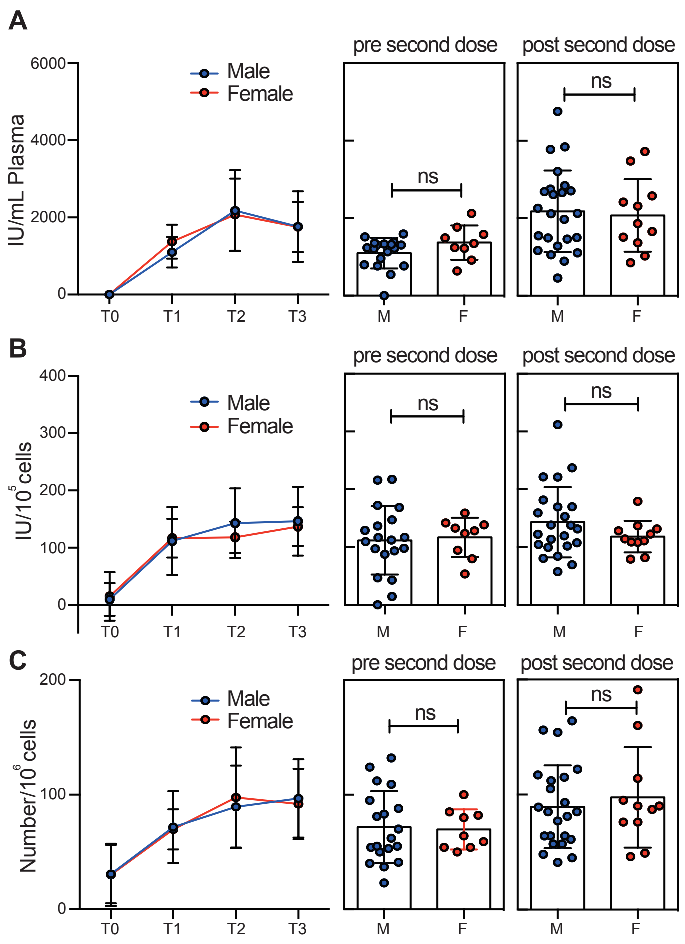

A
IU/mL Plasma
6000
4000
2000
0
Male
Female
T0
T1
T2
T3
pre second dose
post second dose
ns
M
F
B
IU/10^5 cells
400
300
200
100
C
Number/10^6 cells
